# Tailoring plasmon excitations in $\alpha -{\mathcal {T}}_3$ armchair nanoribbons

**DOI:** 10.1038/s41598-021-99596-z

**Published:** 2021-10-18

**Authors:** Andrii Iurov, Liubov Zhemchuzhna, Godfrey Gumbs, Danhong Huang, Paula Fekete, Farhana Anwar, Dipendra Dahal, Nicholas Weekes

**Affiliations:** 1 Department of Physics and Computer Science, Medgar Evers College of the City University of New York, Brooklyn, NY 11225 USA; 2 Department of Physics and Astronomy, Hunter College of the City University of New York, 695 Park Avenue, New York, NY 10065 USA; 3 Donostia International Physics Center (DIPC), P de Manuel Lardizabal, 4, 20018 San Sebastian, Basque Country Spain; 4 Air Force Research Laboratory, Space Vehicles Directorate, Kirtland Air Force Base, Albuquerque, NM 87117 USA; 5 Center for High Technology Materials, University of New Mexico, 1313 Goddard SE, Albuquerque, NM 87106 USA; 6 US Military Academy at West Point, 606 Thayer Road, West Point, NY 10996 USA; 7 Lawrence Berkeley National Laboratory, 1 Cyclotron Road, Berkeley, CA 94720 USA; 8 Texas Center for Superconductivity and Department of Physics, University of Houston, Houston, TX 77204 USA; 9 Department of Information Systems and Cybersecurity, Grove School of Engineering, The City College of New York, 275 Convent Avenue, New York, NY 10031 USA

**Keywords:** Electronic properties and materials, Structure of solids and liquids, Electronic properties and devices

## Abstract

We have calculated and investigated the electronic states, dynamical polarization function and the plasmon excitations for $\alpha -{\mathcal {T}}_3$ nanoribbons with armchair-edge termination. The obtained plasmon dispersions are found to depend significantly on the number of atomic rows across the ribbon and the energy gap which is also determined by the nanoribbon geometry. The bandgap appears to have the strongest effect on both the plasmon dispersions and their Landau damping. We have determined the conditions when relative hopping parameter $\alpha$ of an $\alpha -{\mathcal {T}}_3$ lattice has a strong effect on the plasmons which makes our material distinguished from graphene nanoribbons. Our results for the electronic and collective properties of $\alpha -{\mathcal {T}}_3$ nanoribbons are expected to find numerous applications in the development of the next-generation electronic, nano-optical and plasmonic devices.

## Introduction

Graphene nanoribbons (GNRs) are now one of the most intensively investigated two-dimensional (2D) materials in the field of low-dimensional nanoelectronic devices. This is due to their sensitive dependence on the width and nature of their the edges of the nanoribbons. They also display a number of fundamental physical and technologically promising properties, as well as some unique and unprecedented physical phenomena in comparison with corresponding bulk materials^1^. These includes exotic and non-trivial topological electronic states (Majorana fermions)^2,3^, spin-momentum locked and correlated transport channels, and arrays of plasmonic nano-antennas^4^. In addition, specific electronic quantum phases could be created at junctions of armchair nanoribbons^5^. Transport of charge carriers was studied in networks of armchair nanoribbons, and the possibility for creating a reproducible field-effect transistor with much higher carrier mobility was also demonstrated^6^. Furthermore, plasmonics is playing a major role in designing low-dimensional optical devices since the localized field induced by collective excitations could be closely confined within a nano-size ribbon, resulting in a distinct plasmon mode accompanied by a huge enhancement in the surrounding optical field^7–10^. Specific types of plasmon excitation in nanoribbons^11^ acquire some important and sometimes unexpected features and applications in sensing and nano-imaging^12–14^. Similarly, the plasmons were extensively studies in graphene^15,16^ and other bulk Dirac materials. After more than a decade of uninterrupted efforts^17–24^, many-body theory on low-dimensional materials has finally been developed and becomes a huge resource for in-depth exploration of plasmon dynamical properties. This success is mainly attributed to accurately calculating the dynamical polarization function^25^, which is also related to the effect of static screening on transport properties of electrons.

From a physical point of view, we expect all nanoribbon electronic properties will depend strongly on their width and type of termination^26,27^ as well zigzag^28,29^ and armchair^30–32^. In practice, the studies of quantum size-effect and nonlocality of plasmons, dielectric and optical responses in both nanoribbons^33^, and nanodisks have already revealed a substantial plasmon broadening, much larger for zigzag in comparison with the armchair case^34^. Meanwhile, a tunable bandgap, which is not present for graphene but could be produced by applying an optical field^35–37^, is desirable for most semiconductor devices and depends on the width of armchair nanoribbon^27,32,38^. Consequently, such a bandgap could be adjusted by employing armchair GNRs with various numbers of atomic cells across a ribbon.

Electronic and collective properties of a GNR could be used for high-frequency electronics, nano-scale circuit fabrication and production, of various kinds of very-long-wavelength sensing, and ultrafast electric and light modulations^39^. While low-loss graphene is technologically regarded as a promising replacement for high-frequency plasmonic materials like metals, nanoribbons have become especially attractive since a very strong, induced electric field resulting from resonant plasmons can be sustained across a narrow spatially-confined channel, and furthermore, this resulting electric field could be modified by patterned electrostatic gating^40–42^.

It is important to point out that efficient, reliable and affordable techniques for fabrication of nanoribbons with a given width have already become accessible through chemical vapor deposition^43–45^, in addition to earlier atomically-precise bottom-up fabrication^46^ using chemically or lithographically unzipping a carbon nanotube^47^.

From another perspective, among all newly emergent and recently discovered 2D nanostructures^48,49^, the $\alpha -{\mathcal {T}}_3$ model represents one of the most unusual and promising materials. Its atomic structure consists of a hexagonal-honeycomb lattice as in graphene plus an atom located at the center of each hexagon, i.e. a hub (or *H*) atom. The interaction strength, resulting from electron hopping integral between *H* and either remaining *A* or *B* rim atom, differs from that between nearest-neighbor rim atoms of the hexagon. In this case, the relative hopping parameter $\alpha = t_{\mathrm{hub-rim}}/t_{\mathrm{rim-rim}}$ varies only between 0 and 1, where $\alpha = 0$ corresponds to graphene with a completely detached set of hub atoms, whereas $\alpha = 1$ refers to the dice lattice. Therefore, such an $\alpha -{\mathcal {T}}_3$ model is regarded as an interpolation between graphene and a dice lattice. In reality, a number of $\alpha -{\mathcal {T}}_3$ materials have already been fabricated successfully^50^. The $\alpha -{\mathcal {T}}_3$ lattice structure leads to a pseudospin-1 Dirac-Weyl Hamiltonian, and the resulting metallic (gapless) low-energy band structure involves a Dirac cone as well as an additional dispersionless flat band. This flat band makes the $\alpha -{\mathcal {T}}_3$ topologically distinguished from any other Dirac materials and appears very to be stable and robust against the presence of external fields^51–53^, or a disorder. Another noticeable effects of this flat band appears as a reduced electron mobility in $\alpha -{\mathcal {T}}_3$ material due to the zero group velocity $\backsimeq d\varepsilon ({\varvec{k}}_\Vert )/dk_i$ associated with this flat band for $i=1,\,2$, as well as new physics features for plasmon excitations and their Landau damping^22^.

A bandgap could also be generated in a dice lattice^54,55^, similar to the situation for graphene. Recently, Dirac semimetals also showed some interesting electronic properties^56,57^, although they closely resemble but not completely the same as those of $\alpha -{\mathcal {T}}_3$. As a matter of fact, the unique electronic band structure of the $\alpha -{\mathcal {T}}_3$ model has proven to be responsible for its unusual electronic, optical, collective, magnetic and topological properties^58–77^ which have been investigated extensively over the last several years.

In spite of the fact that $\alpha -{\mathcal {T}}_3$ materials were only discovered recently, there has just been a handful of crucial publications on the subject of nanoribbons made from such materials. Group velocities and current distributions in these ribbons were studied in Ref.^78^ with both armchair and zigzag terminations in the presence of a magnetic field. Importantly, a comprehensive investigation on dice lattice with $\alpha = 1$ indicated a difference in electronic states compared to zigzag graphene nanoribbons^79^. Moreover, electronic states under a magnetic field were calculated in Ref.^80^ for both armchair and zigzag configurations. Meanwhile, mean-field computations on effects due to strain^81^ hsvr revealed a phase transition from antiferromagnetic to ferromagnetic behavior with increasing $\alpha$ value in analogy with the paramagnetic transition in bulk $\alpha -{\mathcal {T}}_3$^82,83^, and this ferromagnetic ordering in dice ribbons was further explored in Ref.^84^. For $\alpha -{\mathcal {T}}_3$ materials, a valley dependence in both bulk^51^ and nanoribbons^85^ is of the highest interest. Most previous theoretical papers on GNR plasmonics deal with a particular case having semi-metallic gapless energy bands and low-level electron doping, for which an approximate analytic calculation for plasmon dispersion could be performed in the long wavelength limit^26,86,87^. Although such simplified approaches are considered realistic and important, they are very limited because both the Dirac Hamiltonian and ${\varvec{k}}\cdot {\varvec{p}}$ model only work well for wide nanoribbons in which several subbands are populated even for moderate doping levels. However, an important case with a finite bandgap has seldomly received enough attention even for earlier studies on graphene nanoribbons. In view of this, the primary focus of our paper is on studying the dependence of plasmon dispersion and their damping on nanoribbon width, which determines the splitting gap between energy subbands, electron doping density, and especially on the relative hopping parameter $\alpha$.

## Phase-dependent electronic states in nanoribbons

Our starting point here is a calculation of both the wave functions and low-energy dispersion of electrons in an $\alpha - {\mathcal {T}}_3$ nanoribbon associated with a bulk pseudospin-1 Dirac-Weyl Hamiltonian^22^

1 $$\begin{aligned} {\mathbb {H}}_b^{\tau ,\,\phi }({\varvec{k}}) = \gamma _0 \left\{ \begin{array}{ccc} 0 &{}\quad k^\tau _- \, \cos \phi &{}\quad 0 \\ k^\tau _+ \, \cos \phi &{}\quad 0 &{}\quad \, k^\tau _- \, \sin \phi \\ 0 &{}\quad k^\tau _+ \, \sin \phi &{}\quad 0 \end{array} \right\} \, . \end{aligned}$$

**Figure 1:**
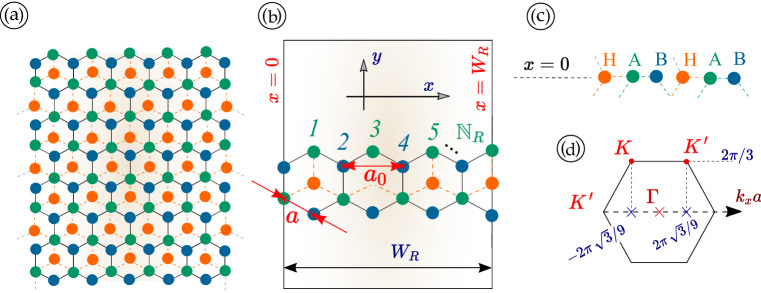
Schematic illustrations for three sublattices ($A,\,B,\,H$) of $\alpha - {\mathcal {T}}_3$ armchair nanoribbons. Panels (**a**) and (**b**) depict representations of a nanoribbon with armchair termination for defining the width $W_R$ of a nanoribbon with inter-atom distance *a* and lattice constant $a_0$. Panel (**c**) demonstrates the edge-row configurations and transverse boundary conditions along the *x* axis for an armchair edge of $\alpha - {\mathcal {T}}_3$ or a dice lattice, where the letters $H,\,A,\,B$ label hub and hexagonal-rim atoms of two sublattices. Panel (**d**) illustrates the first Brillouin zone and the wave vectors ${\varvec{K}}$ and ${\varvec{K}}'$ of two valleys.

In this notation, $\gamma _0=\hbar v_F/\sqrt{2}$, $k^\tau _\pm = \tau k_x \pm i k_y$ depends on the valley index $\tau$ for two non-equivalent *K* and $K^\prime$ valleys. We emphasize that we confine our attention to low-energy states of electrons near these valleys with separation $\delta K_x=K-K'= 4\pi /\sqrt{3}\,a_0$ and $\delta K_y = 0$ where $a_0$ is the lattice constant, as presented in Fig. 1d. For this, the relative hopping parameter $\alpha$ is related to the geometrical phase $\phi$ in Eq. (1) (also called Berry phase sometimes although the real Berry phase is $\pm \pi \cos (2\phi )$ for the conical bands and ${\mp } 2 \pi \cos (2\phi )$ for the flat band of $\alpha - {\mathcal {T}}_3$)^51,88^ by $\alpha = \tan \phi$. As shown in Fig. 1a and b, $a_0 = 0.142\,$nm represents the lattice constant (bond length between two nearest identical atoms, e.g., *B*-to-*B*) and $a = a_0/\sqrt{3}$ is the side length of a hexagon.

In bulk $\alpha - {\mathcal {T}}_3$, solutions for three low-energy bands are $\varepsilon ^{\sigma }_{\tau , \, \phi }({\varvec{k}}) = \sigma \,\hbar v_F k$ with $\sigma = \pm 1$ labeling the valence (−) and conduction ($+$) bands, respectively, and they are identical to those of graphene. Apart from these two subbands, the Hamiltonian in Eq. (1) yields an additional solution $\varepsilon ^{\sigma =0}_{\tau , \, \phi }({\varvec{k}})\equiv 0$ which represents a dispersionless or flat band. Such general schematics with a lower valence, an upper conduction and a middle flat band are retained for nanoribbons. We also note that all these energy dispersions do not depend on the valley index $\tau$ in contrast to the phases contained in corresponding wave functions.

Chosen finite width and edge termination of a ribbon determine its electronic properties. These include the quantization of electron transverse momentum and the presence of splitting gaps between different electron subbands. The width $W_R$ of a ribbon determines the number of atomic rows ${\mathbb {N}}_R$ across the ribbon through $W_R = (a_0/2)\, ({\mathbb {N}}_R+1) = \sqrt{3}a \, ({\mathbb {N}}_R+1)/2$ as shown in Fig. 1a through Fig. 1c. Here, ${\mathbb {N}}_R$ is the total number of atomic rows including all types (*A*, *B* and *H*) of lattice atoms but *excluding the two boundary rows where the wave function would vanish*. The width of the ribbon shown in Fig. 1b is obviously $3.5 {\mathbb {N}}_R$.

The boundary conditions for the wave function are the same as those for a dice lattice^79^ by requiring that all three sublattice probability currents, including one for *H* atom, disappear at each boundary of a nanoribbon. This gives rise to $\varphi _{\nu }(x)\big |_{x=0}=\varphi '_{\nu }(x)\big |_{x=0}$ and $\varphi _{\nu }(x)\big |_{x=W_R}=\texttt {exp}(i \delta K_x W_R) \, \varphi '_{\nu }(x)\big |_{x=W_R}$, where $\nu = A$, *B* and *H*, $\varphi _{\nu }(x)$ and $\varphi '_{\nu }(x)$ correspond to wave function components belonging to ${\varvec{K}}$ and ${\varvec{K}}'$ valleys, respectively. From the armchair boundary conditions, it is clear that the boundary conditions have mixed the electronic states from both ${\varvec{K}}$ and ${\varvec{K}}'$ valleys, similar to graphene^26^, and therefore both valleys need to be considered.

The energy dispersions for an $\alpha -{\mathcal {T}}_3$ armchair nanoribbon obtained in Sect. I of the Supplementary Information appear to be the same for previously considered limiting cases of graphene (except for the presence of the flat band) and a dice lattice: $\varepsilon ^\sigma _n(k_y) = \sigma \gamma _0\,\sqrt{k_y^2 + \xi _n^{\,2}}$ with $\sigma =0,\,\pm 1$. The dispersion relations look similar to bulk $\alpha -{\mathcal {T}}_3$ but with quantized transverse momentum obtained from the condition

2 $$\begin{aligned} 2\xi _n \, W_R = 2 \pi n + \delta K_x W_R, \quad n = 0,\, \pm 1,\, \pm 2,\, \pm 3,\, \cdots , \end{aligned}$$

which determines the quantized transverse wave number $\xi _n$ and energy subbands. These agree with previous results for graphene^26,89^ ($\alpha = 0$) and a dice lattice^79^ ($\alpha = 1$). The $\alpha$-independent result in Eq. (2) tells us that the subband-energy dispersions $\varepsilon _n(k_y)$ will be the same for both graphene and $\alpha - {\mathcal {T}}_3$ materials except for an additional flat band in the middle, which is similar to the case of two bulk materials. Explicitly, from Eq. (2) we obtain the quantized transverse wave number $\xi _n$ of electrons, given by

3 $$\begin{aligned} \xi _n = \frac{\pi n}{W_R} - \frac{2 \pi }{3 a_0} = \frac{2 \pi }{\sqrt{3}\,a} \,\left( \frac{n}{{\mathbb {N}}_R+1}- \frac{1}{3} \right) = \frac{2 \pi }{3 \sqrt{3} \, a} \, \frac{3 n - {\mathbb {N}}_R - 1}{{\mathbb {N}}_R+1} \, . \end{aligned}$$

**Figure 2:**
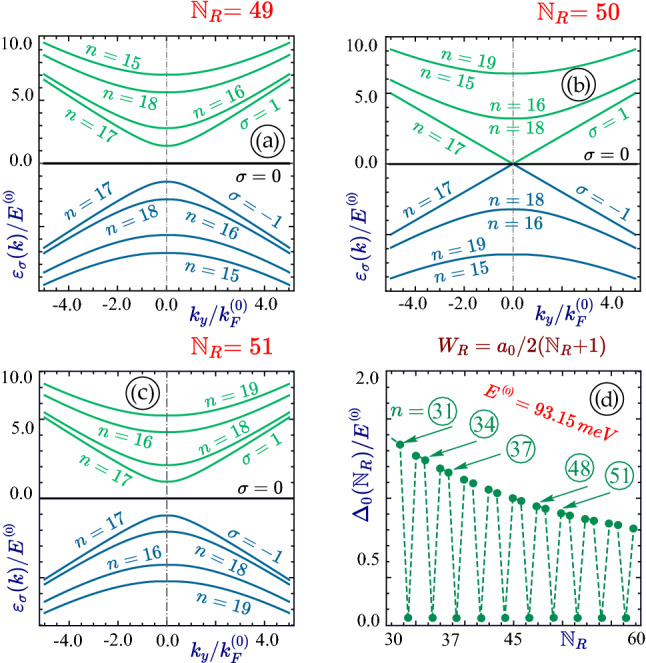
Calculated low-energy subbands $\varepsilon ^\sigma _n(k_y)$ of $\alpha -{\mathcal {T}}_3$ armchair nanoribbons with different widths $W_R$. Panels (**a**) through (**c**) present dispersion relations of some low-energy subbands as functions of the longitudinal wave vector $k_y$ for ribbons with ${\mathbb {N}}_R = 49,\,50,\,51$ atomic rows, respectively. Panel (**d**) shows the calculated half-bandgap $\Delta _0({\mathbb {N}}_R)$ of nanoribbons between the lowest conduction and the middle flat subband for various selected ${\mathbb {N}}_R$ values. In our computations, we set units as $k_F^{(0)} = (\pi /2)\, (10^8 \,$m$^{-1}) = 1.57 \times 10^8 \,$m$^{-1}$, $L_0 = 1/k_F^{(0)} = 6.33\,$nm, $E^{(0)} = \gamma _0\,k_F^{(0)} = 93.15\,$meV and $\Omega _0=1.42 \times 10^{14}\,$Hz.

The obtained dispersions imply that the $n=1$ energy subband may not be the lowest one in contrast to the case for a quantum well. As expected, some of the calculated low-energy subbands $\varepsilon ^\sigma _N(k_y)$ presented in Fig. 2a–c do depend on the quantum number *n* and the number of row atoms $N_R$. Interestingly, the bandgap between the lowest conduction subband and the highest valence subband varies with $W_R$ or ${\mathbb {N}}_R$, becoming finite for $N_R=49$ and 51 but zero for $N_R=50$.

As a matter of fact, the energy gap between the first valence (or conduction) subband and the middle flat band at zero energy, i.e., half of the bandgap, is found to be

4 $$\begin{aligned} \Delta _0({\mathbb {N}}_R) = \frac{2 \pi }{3 \,a_0} \, \left( \frac{\gamma _0}{{\mathbb {N}}_R +1}\right) \, \min \limits _{n\in \{N_\Delta \}}\,(3 n - {\mathbb {N}}_R - 1)\ , \end{aligned}$$

where the minimal value of $3 n - {\mathbb {N}}_R - 1$ for a given ribbon width ${\mathbb {N}}_R$ is assumed to be achieved for integer numbers $n_{\Delta }$, and $\Delta _0({\mathbb {N}}_R)$ becomes zero if ${\mathbb {N}}_R + 1$ becomes divisible by 3. Therefore, $N_0 = ({\mathbb {N}}_R + 1)/3$ enables specifying the lowest pair of metallic subbands which touch each other, and the flat band as well, at the Dirac point. Otherwise, $n_\Delta$ should be the smaller one of following two numbers, i.e., $n_{\Delta }^\pm = {\text {InP}}\left[ ({\mathbb {N}}_R + 1)/3 \right] \pm 1$, where ${\text {InP}}(x)$ is a function by taking the integer part of a rational number *x*. The calculated $\Delta _0({\mathbb {N}}_R)$ as a function of ${\mathbb {N}}_R$ is presented in Fig. 2d, from which we find $\Delta _0({\mathbb {N}}_R)$ does go to zero for a set of selected values of ${\mathbb {N}}_R$ or $W_R$. All the other (higher) subband dispersions are doubly degenerate, as seen in Fig. 2a–c. Meanwhile, we also find that both subband separation and energy gap are greatly enhanced for a narrower ribbon, similar to the case for a quantum well, which makes it difficult to occupy more than one subband for a range of experimentally-accessible doping levels.

The wave function for an armchair nanoribbon takes the form

5 $$\begin{aligned} \Phi ^\phi _{\sigma }(n \, \vert \, x, k_y) = \left\{ \begin{array}{l} \Psi ^{\mathbf{K}, \, \phi }_{\sigma }(n \, \vert \, x, k_y) \\ \Psi ^{\mathbf{K}', \, \phi }_{\sigma }(n \, \vert \, x, k_y) \end{array} \right\} \, . \end{aligned}$$

Each of the two components in Eq. (5) is related to a specific valley ($\tau = \pm 1$) and formally written as

6 $$\begin{aligned} \Psi ^{\tau , \phi }_{\sigma = \pm 1}(n \, \vert \, x, k_y) = \frac{1}{\sqrt{\mathcal{{L}}_0W_R}} \, \varphi ^{\tau , \phi }_{\sigma = \pm 1}(n \, \vert \, x) \, \texttt {e}^{i k_y y} \ , \end{aligned}$$

where $\mathcal{{L}}_0$ is the ribbon length,

7 $$\begin{aligned} \varphi ^{\tau , \phi }_{\sigma = \pm 1}(n \, \vert \, x) = \frac{1}{\sqrt{2}} \left[ \begin{array}{c} \tau \cos \phi \, \texttt {e}^{- i \tau \Theta _n(k_y)} \\ \sigma \\ \tau \sin \phi \, \texttt {e}^{i \tau \Theta _n(k_y)} \end{array} \right] \, \texttt {exp}(i \tau \xi _n x)\ . \end{aligned}$$

and $\Theta _n(k_y)=\tan ^{-1}(k_y/\xi _n)$. It is crucial to notice that only the electron/hole index $\sigma = \pm 1$, not the subband index *n*, determines the ± sign of energy dispersions $\varepsilon ^\sigma _n(k_y)$.

The eigenvalue equation in $\alpha -{\mathcal {T}}_3$ allows for an additional zero-energy solution with for a flat band $(\sigma = 0)$ which is

8 $$\begin{aligned} \Psi ^{\tau , \phi }_{\sigma = 0}(n \, \vert \, x, k_y) = \frac{1}{\sqrt{\mathcal{{L}}_0W_R}} \, \, \varphi ^{\tau , \phi }_{\sigma = 0}(n \, \vert \, x) \, \texttt {e}^{i k_y y} \, , \end{aligned}$$

where

9 $$\begin{aligned} \varphi ^{\tau , \,\phi }_{\sigma = 0}(n \, \vert \, x) = \left[ \begin{array}{c} \left( \tau \zeta _{n} - i k_y \right) \,\sin \phi \\ 0 \\ -\left( \tau \zeta _{n} + i k_y \right) \,\cos \phi \end{array} \right] \, \frac{\texttt {e}^{i \tau \zeta _{n} x}}{\sqrt{\zeta _{n}^2 + k_y^2}} = \left[ \begin{array}{c} \tau \sin \phi \, \texttt {e}^{- i \tau \Theta _n(k_y)} \\ 0 \\ - \tau \cos \phi \, \texttt {e}^{i \tau \Theta _n(k_y)} \end{array} \right] \, \texttt {exp}(i \tau \xi _n x) \ , \end{aligned}$$

in which ${\varvec{K}}$ and ${\varvec{K}}'$ valleys correspond to $\tau = \pm 1$ and $\Theta _n(k_y) = \tan ^{-1}(k_y/\zeta _n)$ and $\zeta _n = \vert \xi _n \vert$ given by Eq. (3).

As far as the obtained electronic states are concerned, we would like to emphasize that the type, structure and dependence of the wave functions in Eqs. (7) and (9) are very similar in nature to those for the bulk material. However, the transverse wave vector $\xi _n$ and the quantum phase $\Theta _n(k_y)$, are now fully quantized (or discrete), and the quantization conditions in Eq. (3) are identical and include the mixing of ${\varvec{K}}$ and ${\varvec{K}}'$ valleys.

## Polarization function, plasmon dispersions and damping

For an ideal graphene nanoribbon without edge defects, by using standard many-body theory, the dielectric-function tensor within the random-phase approximation (RPA) can be generally expressed as

10 $$\begin{aligned} \epsilon ^{\lambda , \,\rho }_{\mu , \nu }(q_y, \omega \, \vert \, E_F, \alpha ) = \delta _{\lambda , \mu }\,\delta _{\rho , \nu } - V_{\mu , \nu }^{\lambda , \rho }(q_y) \,\Pi ^{(0)}_{\mu , \nu }(q_y, \omega \, \vert \, E_F, \alpha )\ , \end{aligned}$$

where $E_F$ denotes the Fermi energy of the system, $q_y$ is the longitudinal transfer wave vector along a nanoribbon and $\omega$ is the angular frequency of a perturbing field. Here, each of the indices $\lambda$, $\rho$, $\mu ,\,\nu$ is a composite one which includes the subband index *i* and band index $\sigma$ for the conduction, valence and flat bands, i.e., $\lambda = \{i,\sigma \}$. The plasmon modes of the structure can be computed from the zero determinant of the dielectric-function tensor in Eq. (10), leading to

11 $$\begin{aligned} \mathcal{{D}}et \, \left[ \epsilon ^{\lambda , \rho }_{\mu , \nu }(q_y, \omega \, \vert \, E_F, \alpha ) \right] = \mathcal{{D}}et \, \left[ \delta _{\lambda , \mu }\,\delta _{\rho , \nu } - V_{\mu , \nu }^{\lambda ,\rho }(q_y) \,\Pi ^{(0)}_{\mu , \nu }(q_y, \omega \, \vert \, E_F, \alpha ) \right] = 0 \ . \end{aligned}$$

The diagonal matrix elements in Eq. (10) are connected to dispersions of individual plasmon modes, while the off-diagonal matrix elements describe the couplings between different plasmon modes.

Within the RPA, the introduced subband polarization function $\Pi ^{(0)}_{\mu , \nu }(q_y,\omega )$ in Eq. (10) is calculated as

12 $$\begin{aligned} \Pi ^{(0)}_{\mu , \nu }(q_y,\omega \, \vert \, E_F, \alpha )=\frac{g_s}{2\pi }\,\int \limits _{1^{\mathrm{st}}\,{\mathrm{BZ}}} dk_y\, \left\{ \frac{ f_0[\varepsilon _{\mu }(k_y)]-f_0[\varepsilon _{\nu }(k_y+q_y)] }{ \hbar (\omega +i\delta ) + \varepsilon _\mu (k_y) - \varepsilon _\nu (k_y+q_y) } \right\} \,{\mathbb {O}}_{\sigma _\mu \leftrightarrow \sigma _\nu }^{m,n}(k_y, q_y \, \vert \, \alpha )\ , \end{aligned}$$

where the integral with respect to wave vector $k_y$ is limited to the first Brillouin zone, $g_s=2$ takes into account the spin degeneracy, $\delta \ll \omega$ represents a homogeneous diagonal-dephasing rate of electrons and $\varepsilon _\mu (k_y)$ is the subband energy, $f_0(x)$ is is the Fermi-Dirac distribution function for thermal-equilibrium electrons at temperature *T* and chemical potential $u_0(T)$. At $T=0$,

13 $$\begin{aligned}&f_0[\varepsilon _\mu (k_y) \, \vert \, u_0(T), T \rightarrow 0]= \left. \left\{ 1+\texttt {exp} \left[ \frac{\varepsilon _\mu (k_y)-u_0(T)}{k_B T}\right] \right\} ^{-1}\right| _{T\rightarrow 0} \rightarrow \delta _{\sigma ,-1} + \delta _{\sigma ,0} +\delta _{\sigma , 1} \,\Theta [E_F - \varepsilon _m (k_y)] \ , \end{aligned}$$

where $E_F$ is the Fermi energy and $\Theta (x)$ is a Heaviside step function.

**Figure 3:**
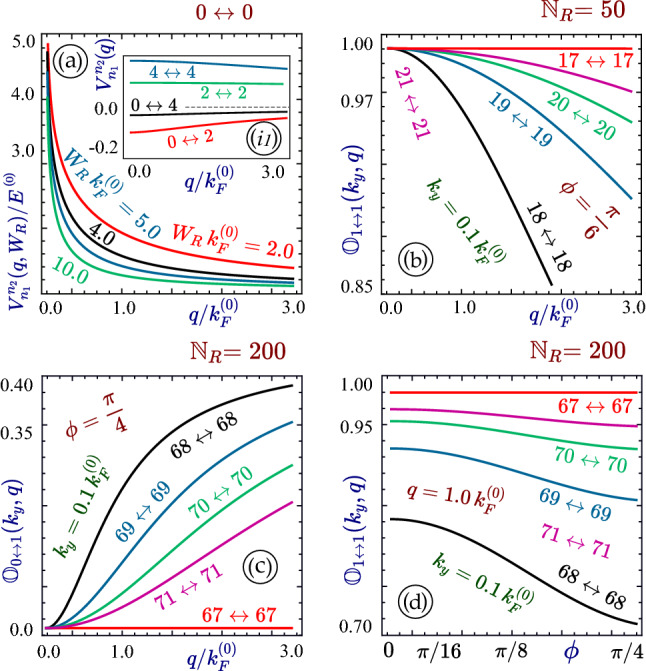
Panel (**a**) shows the Coulomb potential $V_{n_1}^{n_2}(|q_y|W_R)$ with $n_1 = i-j = 0$ and $n_2 = m-n = 0$ as a function of the transfer wave vector $q_y$ for chosen widths $W_R$, and its inset (*i*1) displays the remaining potential elements with nonzero $n_1$ or $n_2$, as labeled. Panels (**b**) through (**d**) present *intra-subband* ($n_1 = n_2$) overlaps ${\mathbb {O}}_{\sigma _1 \leftrightarrow \sigma _2}^{n_1,n_1}(k_y,q_y)$ for various types of * intra * ($0 \leftrightarrow 1$) and *inter-band* ($1 \leftrightarrow 1$) transitions as functions of $q_y$ in (**b**), (**c**) and phase $\phi$ in (**d**). Here, ${\mathbb {N}}_R$ is set as $50,\,50,\,200$ in (**b**), (**d**). Other parameters are the same as those in Fig. 2.

Next, since the mirror symmetry between conduction and valence subbands is retained in our system, as it is for graphene nanoribbons, it guarantees that the orbital part of the wave function will not depend on band selection. Consequently, such a unique property greatly simplifies the Coulomb interaction $V_{\mu , \nu }^{\lambda ,\rho }(q_y)$ employed in Eq. (10), leading to

14 $$\begin{aligned} V^{j,m}_{j',m'}(q_y)=\frac{e^2}{2\pi \epsilon _0\epsilon _b}\,\int \limits _0^1 du\,\int \limits _0^1 du'\,\cos [\pi (j-m)u]\,\cos [\pi (j'-m')u']\,K_0(|q_y|W_R|u-u'|)\equiv V^{j-m}_{j'-m'}(q_y)\ , \end{aligned}$$

which is significant only for $j-j'=0$ and $m-m'=0$, as demonstrated in Fig. 3a. Moreover, the Coulomb interaction in Eq. (14) is also independent of the phase $\phi$, and therefore, remains the same for all $\alpha -{\mathcal {T}}_3$ materials including graphene^89^. In the long-wavelength limit $q_y \rightarrow 0$, the Bessel function $K_0(x)$ of the second kind diverges as $-\log (x)$, corresponding to $\backsimeq 1/\sqrt{q^2_x+q^2_y}$ behavior for the 2D case. In this case, the Coulomb-interaction matrix elements connect two initial and two final states with the same band and subband indices, leading to one for intra-subband excitation only, i.e.

15 $$\begin{aligned} \epsilon _{\mu ,\nu }(q_y, \omega \, \vert \, E_F, \alpha )\equiv \epsilon ^{\mu ,\nu }_{\mu ,\nu }(q_y, \omega \, \vert \, E_F, \alpha ) \approx \delta _{\sigma _\mu , \sigma _\nu } \delta _{m,n} - V_{0}^0(q_y) \,\Pi ^{(0)}_{\nu , \nu }(q_y, \omega \, \vert \, E_F, \alpha )\ , \end{aligned}$$

which is a regular 2D matrix ($3N \times 3N$), where $\nu =\{n,\sigma _\nu \}$ and *N* is the total number of subbands taken into consideration.

Finally, the determinant ${\mathcal {D}}et$ and the trace ${\mathcal {T}}r$ of the matrix in Eq. (15) are (see Sect.  II of our Supplementary Information) connected by

16 $$\begin{aligned} {\mathcal {D}}et \left[ \epsilon _{\mu \nu }(q_y, \omega ) \right] = 1 - V_{0}^0(q_y) \, \sum \limits _{\nu } \Pi ^{(0)}_{\nu , \nu }(q_y, \omega \, \vert \, E_F, \alpha ) = {\mathcal {T}}r \left[ \epsilon _{\mu \nu }(q_y, \omega ) \right] - 3 N + 1 \ , \end{aligned}$$

which was also employed in Ref.^89^ for graphene. In our calculation, the actual polarization function is obtained as the sum of ten lowest subbands around $n_0$.

As a last step, we turn to calculate the wave-function overlaps (or prefactors) ${\mathbb {O}}^{\,n,n'}_{\pm 1,0 \leftrightarrow \pm 1,0} (k_y,q_y)$ introduced in Eq. (12) for the polarization function. For $\alpha -{\mathcal {T}}_3$ nanoribbons, it is explicitly defined by ${\mathbb {O}}^{\,n,n'}_{\pm 1,0 \leftrightarrow \pm 1,0} (k_y, q_y) \equiv \left| \langle \Phi ^\phi _{\sigma }(n \, \vert \, x,k_y) \, \vert {\texttt {e}}^{i q_x x} \vert \,\Phi ^\phi _{\sigma }(n' \, \vert \, x,k_y + q_y) \rangle \right| ^2$ where $\sigma = 0,\,\pm 1$ and the complete wave functions $\Phi ^\phi _{\sigma }(n \, \vert \, x,k_y)$ and $\Phi ^\phi _{\sigma }(n' \, \vert \, x,k_y + q)$ have already been given by Eq. (5).

Using earlier wavefunctions (5), we get ${\mathbb {O}}^{\,n,n'}_{\pm 1 \leftrightarrow 1} (k_y,q_y) = \frac{1}{4}\, \left[ 1 \pm \cos [\Theta _{n,n'}(k_y,q_y)] \right] ^2 + \frac{1}{4} \, \cos ^2 (2 \phi ) \sin ^2 [\Theta _{n,n'}(k_y,q_y)]$ and ${\mathbb {O}}^{\,n,n'}_{0 \leftrightarrow 1} (k_y,q_y) = \frac{1}{2}\, \sin ^2 (2 \phi ) \, \sin ^2 [\Theta _{n,n'}(k_y,q_y)]$ which are the same as our obtained result for bulk $\alpha -{\mathcal {T}}_3$^90^ since ${\mathbb {O}}^{\,n,n'}_{\pm 1,0 \leftrightarrow \pm 1,0} (k_y, q_y)$ does not depend on the valley index $\tau$ while the wave function in Eq. (5) for an armchair nanoribbon is just a combination of states from these two valleys. Therefore, the overlap can be regarded as an average between two inequivalent valleys. On the other hand, the wave functions in Eq. (5) do depend on the band indices so that the results are different, corresponding to various types of transitions (i.e., from and to valence, flat, and conduction bands). For graphene with $\alpha = 1$, we easily verify that ${\mathbb {O}}^{\,n,n'}_{\pm 1 \leftrightarrow \pm 1} (k_y,q_y) = (1 \pm \cos [\Theta _{n,n'}(k_y,q_y)])/2$ as obtained in Ref.^26^. For the opposite limiting case of a dice lattice, however, we have ${\mathbb {O}}^{\,n,n'}_{\pm 1 \leftrightarrow \pm 1} (k_y,q_y) =(1 \pm \cos [\Theta _{n,n'}(k_y,q_y)])^2/4$.

Here, we would like to emphasize that the overlaps discussed above result in crucial difference between plasmons in nanoribbons and bulk $\alpha -{\mathcal {T}}_3$ materials. The angle $\Theta _{n,n'}(k_y,q_y)$ between the wave vectors $k_y$ and $k_y + q_y$ (i.w., either 0 or $\pi$) is quantized in a nanoribbon due to $W_R$-dependent quantization of allowable values for the transverse wave number $\xi _{n\,(n')}$, while the longitudinal wave numbers $k_y$ and $k_y + q_y$ are always directed along the $y-$axis. Since the largest contribution to the polarization function comes from several of the lowest subbands with the minimal values for $\xi _{n\,(n')}$, the resulting angles $\Theta _{n,n'}(k_y,q)$ are often close to 0 or $\pi$ so that a large number of relevant overlaps are equal to either 1 or 0, especially in the long wavelength limit with $q_y\rightarrow 0$. This nanoribbon-specific feature is drastically different from that of a bulk material.

## Results and discussion

We begin our calculations for an armchair $\alpha -{\mathcal {T}}_3$ nanoribbon with metallic (gapless) bandstructure. The lowest and the next subbands above are assumed well separated from each other. We adopt a single-subband model for calculating the polarization function previously employed for graphene nanoribbon^26,86,87^. Such a model could be considered sufficiently accurate for a narrow ribbon with low electron doping $E_F$.

The transverse wave vector $\xi _n$ at the Fermi surface becomes negligibly small, and then the angle $\theta _n$ associated with the wave vector $\{\xi _n, k_y\}$ becomes

17 $$\begin{aligned} \theta _n(k_y)\approx \left\{ \begin{array}{ll} -\pi /2 &{}\quad {\text {if}} \quad k_y < 0, \\ \pi /2 &{}\quad {\text {if}} \quad k_y > 0 \ . \end{array} \right. \end{aligned}$$

The angle $\Theta _{n,n'} (k_y,q_y)$ between two states $\{\xi _n, k_y\}$ and $\{\xi _n, k_y + q_y\}$ is

18 $$\begin{aligned} \Theta _{n,n'} (k_y,q_y) = \left\{ \begin{array}{ll} -\pi &{}\quad {\text {if}} \quad k_y> 0 \,\,{\text {and}}\,\, k_y + q_y< 0 , \\ 0 &{}\quad {\text {if}} \quad k_y(k_y + q_y)> 0 , \\ \pi &{}\quad {\text {if}} \quad k_y < 0 \,\,{\text {but}}\,\, k_y + q_y > 0 . \end{array} \right. \end{aligned}$$

In all cases, this leads to $\sin [\Theta _{n,n'}(k_y,q_y)] = 0$, ${\mathbb {O}}^{\,n,n'}_{0 \leftrightarrow 1} (k_y,q_y) = 0$, and ${\mathbb {O}}^{\,n,n'}_{\pm 1 \leftrightarrow 1} (k_y,q_y) =(1 \pm \cos [\Theta _{n,n'}(k_y,q_y)])^2/4$ is either 0 or 1 due to

19 $$\begin{aligned} {\mathbb {O}}^{\,N_0,N_0}_{\sigma \leftrightarrow \sigma '} (k_y, k_y + \beta q) = \delta _{\sigma \sigma ', \,{\text {sign}} [ k_y \cdot ( k_y + \beta q)] } \, , \end{aligned}$$

where $$\sigma $$, $$\sigma ' \ne 0$$ and $$\beta = \pm 1$$.

For $T=0$, the Fermi distribution functions are reduced to (13), and the polarizability could be presented as

20 $$\begin{aligned} \Pi ^{(0)}(q,\omega ) = - \chi ^{(-)}_{\infty }(q, \omega ) + \sum \limits _{\beta = \pm 1} \chi ^{(\beta )}_{E_F}(q, \omega ) \, , \end{aligned}$$

where the first term corresponds to zero doping (which is possible only for graphene with $\phi = 0$ due to the momentum degeneracy in the flat band) and is equal to

21 $$\begin{aligned} \chi ^{(-)}_{\infty }(q, \omega ) = g_s \sum \limits _{\beta = \pm 1} \, \int \frac{dk_y}{2 \pi } \, \frac{{\mathbb {O}}^{\,N_0,N_0}_{- 1 \leftrightarrow + 1} (k_y, k_y + q)}{ \varepsilon _{N_0}(k_y) + \varepsilon _{N_0}(k_y + q) + \beta \, \omega } \, . \end{aligned}$$

The expansion of (20) looks similar to that for bulk graphene^19^. However, there is a crucial difference, i.e., the integration is performed only over one variable $k_y$ (there is no angular integral). The two terms with $k \pm q$ in the sum in Eq. (21) are substantially different and cannot replace one another. For that reason, there is no unified analytic expression for all three terms in Eq. (20). The two remaining terms of Eq. (20) which appear only for finite doping are

22 $$\begin{aligned} \chi ^{(\rho = \pm )}_{E_F}(q, \omega ) = g_s \sum \limits _{\beta = \pm } \int \frac{dk_y}{2 \pi } \, {\mathbb {O}}^{\,N_0,N_0}_{\rho \, \leftrightarrow + 1} (k_y, k_y + \beta q) \, \frac{\Theta \left[ E_F - \varepsilon _{N_0}(k_y) \right] }{ \varepsilon _{N_0}(k_y) - \rho \, \varepsilon _{N_0}(k_y + \beta q) + \beta \, \omega } \end{aligned}$$

The zero-doping term in Eq. (21) is immediately evaluated as

23 $$\begin{aligned} \chi ^{(-)}_{\infty }(q, \omega ) = - \frac{2 \gamma _0}{\pi } \, \frac{q^2}{\omega ^2 - (\gamma _0 q)^2} \, , \end{aligned}$$

and the other two terms in the expansion (20) are

24 $$\begin{aligned} \chi ^{(-)}_{E_F}(q, \omega ) = g_s \sum \limits _{\beta = \pm } \, \int \frac{dk_y}{2 \pi } \, \Theta \left[ \frac{E_F}{\gamma _0} - \vert k_y \vert \right] \, \Theta [-\beta k_y (k_y + \beta q)] \, (\gamma _0 q + \beta \omega )^{-1} = - \frac{2 \gamma _0 q}{\pi } \, \frac{{\text {Min}}(q, k_F)}{\omega ^2 - (\gamma _0 q)^2} \, . \end{aligned}$$

and

25 $$\begin{aligned} \chi ^{(+)}_{E_F}(q, \omega ) = \frac{2 \gamma _0}{\pi } \, \frac{k_F \, q}{\omega ^2 - q^2} \, \left\{ 1- \left( 1 - \frac{q}{k_F} \right) \, \Theta [k_F - q] \right\} \, , \end{aligned}$$

which is equivalent to

26 $$\begin{aligned} \chi ^{(+)}_{E_F}(q, \omega ) = \frac{2 \gamma _0 q}{\pi } \, \frac{{\text {Min}}(q, k_F)}{\omega ^2 - (\gamma _0 q)^2} = - \chi ^{(-)}_{E_F}(q, \omega ) \, , \end{aligned}$$

which was demonstrated in Sect. III of the Supplementary Information. We have found that the two terms corresponding to finite doping always cancel each other. This is equivalent to finite-temperature behavior discussed in Ref.^87^ but differs from the results of Ref.^86^ for graphene. Our final result in Eq. (21) for the polarization function shows no dependence on $\alpha$ and is the same as for GNR’s^86,89^. The plasmon mode $\Omega _p(q_y)$ in the long-wavelength limit is immediately obtained as^89^

27 $$\begin{aligned} \Omega _p(q_y) = \frac{\gamma _0}{\hbar }\,\sqrt{ - q_y^2 \, \ln (q_y \, W_R)} \ , \end{aligned}$$

which also does not depend on the doing level $E_F$ and parameter $\alpha$ due to the absence of such dependence in Coulomb matrix elements (14).

In most realistic cases, however, the cross momentum $\xi _n \ne 0$ and all the wave function overlaps are finite and depend on $q_y$. This occurs if $\Theta _{n,n}(k_y,q_y)$ differs from 0 and $\pi$ which is achieved for a finite gap or $\xi _n$ disregarding the ribbon width $W_R$. However, for large $W_R$, it approaches bulk $\alpha -{\mathcal {T}}_3$ since the change in $\xi _n$ with *n* decreases ($\propto 1/W_R$). In this case, the dependence on $q_y$ in the range of $q_y/k_F\le 1$ also varies with $k_y$ and its dependence in $\xi _n(k_y)$, as seen in Fig. 3.

The difference between graphene and other types of $\alpha -{\mathcal {T}}_3$ lattices arises from transitions from/to the flat band^22^. Since the $0 \leftrightarrow 1$ overlap contains the factor $\sin ^2 (2 \phi ) \, \sin ^2 [\Theta _{n,n}(k_y,q_y)]$, it will be suppressed as $\alpha \backsimeq 0$ or $\phi \backsimeq 0$, i.e., for all materials resembling graphene and small $\xi _n$ in a wide ribbon. Consequently, we conclude that the $\alpha$ dependence of overlap in the range $q_y/k_F\ll 1$ becomes appreciable only for a sufficiently wide ribbon, as demonstrated in Fig. 3b through Fig. 3c. On the other hand, if $q_y/k_F\backsim 1$ is achieved, the $\alpha$ dependence of the overlap is noticeable for all ribbon widths (e.g., see Fig. 3d).

**Figure 4:**
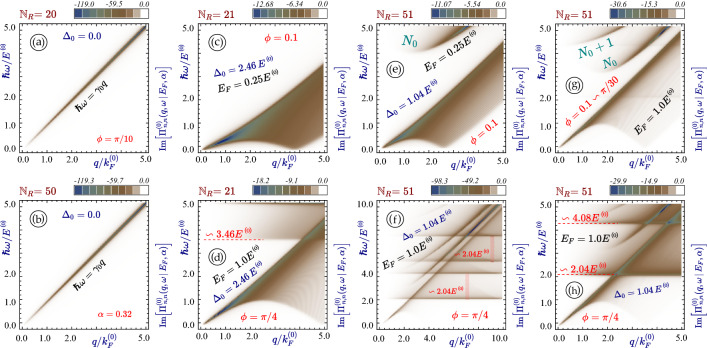
Single-particle excitation spectrum in $\alpha -{\mathcal {T}}_3$ nanoribbons. All panels (**a**) through (**h**) present density plots as functions of frequency $\omega$ and $q_y$ for the imaginary part of the non-interacting *intra-subband* polarization function $\Pi ^{(0)}_{n,n}(q_y, \omega \, \vert \, E_F, \alpha )$ with chosen widths ${\mathbb {N}}_R = 20$, 21, 50 and 51. The material bandgap $\Delta _0/E^{(0)}$ determined by ${\mathbb {N}}_R$ is equal to zero in (**a**), (**b**) but 2.46 in (**c**), (**d**) and 1.04 in (**e**)–(**h**), respectively. The relative hopping parameter $\alpha$ was chosen as $\alpha \backsim \phi = 0.1$ for panels (**c**), (**e**) and (**g**), $\alpha =0.32$ (or $\phi =\pi /10$) for (**a**) and (**b**) and $\phi = \pi /4$ (a dice lattice) for the remaining plots (**d**), (**f**) and (**h**).

The imaginary part of the intra-subband polarization function for armchair nanoribbon is presented in Fig. 4. If there is no bandgap (metallic case, see Fig. 4a,b), the single-particle excitation spectrum is only a strong peak around the main diagonal $\hbar \omega = \gamma _0 q_y$. This dissipation is slightly enhanced with increasing ribbon width since more electron subbands are occupied for the same doping value.

A very different situation occurs if ${\mathbb {N}}_R$ is chosen such that the gap $\Delta _0 > 0$. The previously narrow single-particle excitation region now occupies a sizable area in the $q_y$ - $\omega$ plane, as shown in panels (*a*) through (*h*) of Fig. 4. It is split into two or more broad sub-domains bounded by semi-parabolic edges and separated by a gap, and the whole picture becomes qualitatively similar to bulk gapped graphene^18^ or $\alpha -{\mathcal {T}}_3$^72^. The lower domain corresponds to the intra-band (conduction-to-conduction) excitations while the upper one - to the inter-band (valence-to-conduction and vice versa) ones. Meanwhile, the magnitudes of dissipation within all the intra- and inter-band damping regions are modified greatly by the ribbon width (compare panels (*c*) and (*d*)).

Another area of finite ${\text {Im}} \left[ \Pi ^{(0)} (q, \omega ) \right]$ is uniformly distributed above the Fermi level. It arises due to inter-band electron transitions between the valence and flat bands. It is seen in all cases (panels (*d*), (*f*) and (*h*)) when $\alpha > 0$. We conclude that the difference between $\alpha -{\mathcal {T}}_3$ and graphene ribbons is substantial for any ribbon width if a finite gap is present. The lower boundary of this area is found at frequency $\hbar \omega \backsimeq E_F + \Delta _0$ which corresponds to the energy separation between the flat band (zero-energy level) and the actual Fermi level above the gap. We also noticed that $0 \leftrightarrow 1$ particle-hole mode consists of multiple separate sub-areas corresponding to the transitions from and to the various discrete energy subbands (see panels (*f*) and (*h*) shown for the same nanoribbon but in different ranges of $\omega$ and *q*).

**Figure 5:**
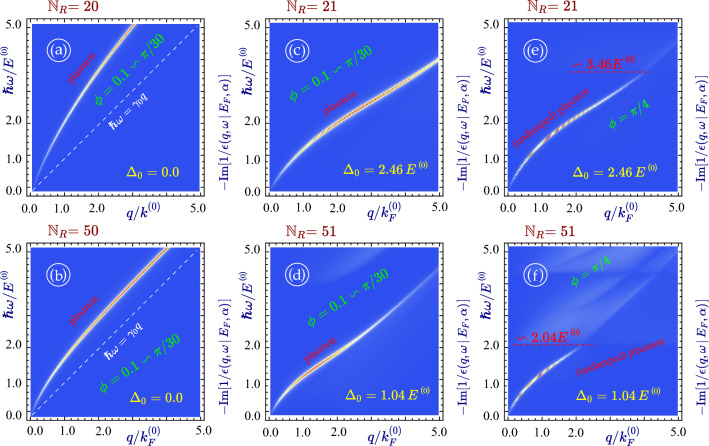
Plasmon excitations in $\alpha -{\mathcal {T}}_3$ nanoribbons. All panels (**a**) through (**d**) display density plots of the spectral loss function ${\mathbb {S}}(q_y, \omega \, \vert \, E_F, \alpha ) = - {\text {Im}}\left[ 1/\epsilon (q_y, \omega \, \vert \, E_F, \alpha )\right]$ as functions of $\omega$ and $q_y$ with ${\mathbb {N}}_R = 20$, 21, 50 and 51. The undamped plasmon excitations correspond to sharp peaks of the loss function (ideally, ${\mathbb {S}} \rightarrow \infty$), therefore, the colorbars were not added to these panels. The values of the material bandgap are determined solely by the width of a nanoribbon and are exactly the same as we have for Fig. 4. The plasmon dispersions in (**a**) and (**b**) are related to metallic electronic spectra (**a**) and (**c**). However, they are to semiconducting ones in all other panels. Parameter $\alpha$ was chosen as $\pi /4$ (a dice lattice) for the lowest panels (**e**) and (**f**) but $\alpha \backsim \phi =0.1$ for all the other plots. Panel (**f**) shows the same situation as (**h**) but for a larger (doubled) ranges of *q* and $\omega$.

The plasmon frequencies for armchair nanoribbon with chosen ribbon widths ${\mathbb {N}}_R$ and bandgaps $\Delta _0$ is shown in Fig. 5. as the peaks of the loss function ${\mathbb {S}}(q_y, \omega \, \vert \, E_F, \alpha )$. The loss function, or the spectral function, is defined as ${\mathrm{Im}}[1/\epsilon (q_y,\omega )]$ through our calculated dielectric function $\epsilon (q_y,\omega )$, which has been utilized for graphically plotting ${\mathrm{Re}}[\epsilon (q_y,\omega )]=0$ or maximum values of ${\mathrm{Im}}[1/\epsilon (q_y,\omega +i\delta )]$ with $\delta /\omega \ll 1$ within the $(q_y,\omega )$-plane^91,92^. On the other hand, the physically-defined loss function ${\mathrm{Im}}[\mathcal{{S}}(q_\Vert ,\omega )]$ has been widely employed for describing power absorption of electron beam either parallel or perpendicular to the surface of an electronic system, where $\mathcal{{S}}(q_\Vert ,\omega )$ is the surface response function, related to the inverse dielectric-function matrix $\overleftrightarrow {\epsilon }^{-1}(q_\Vert ,\omega )$ of electronic system^93,94^.

As $\Delta _0=0$, for a relatively narrow ribbon in Fig. 5a, the group velocity of intra-subband plasmon mode is increased greatly in comparison with a wider ribbon in Fig. 5c. When $\Delta _0$ becomes finite, the group velocity of intra-subband plasmon mode is reduced significantly, as shown in panels (*b*) and (*d*), similar to $\backsimeq \left( 1 - \Delta _0^2/E_F^2 \right)$ dependence for the plasmon group velocity in bulk. In comparison with panel (*b*), a wider ribbon in panel (*d*) further develops a unique concave-to-convex feature in plasmon dispersion, resembling bulk material and meanwhile acquiring an increased dissipation at this “crossing” point. For finite $\alpha$, the plasmon becomes strongly damped at the Fermi level (above the bandgap), as shown in panels (*e*) and (*f*) of Fig. 5. Figures 4 and 5 are presented in a way that in most cases the particle-hole modes and the plasmons are demonstrated for the same nanoribbons (panels (*a*) and (*b*) in both graphs and some others).

The dielectric function is defined as determinant of the dielectric tensor (10), where the Coulomb potential was defined in Eq. (14) as $V_0^{0}(q_y)\backsim e^2/(2 \epsilon _0 \epsilon _r)\,K_0(|q_y|W_R) \backsimeq -2 \pi \alpha _\epsilon \,\ln (|q_y|W_R) \sim 2\pi \alpha _\epsilon /(|q_y|W_R)$. The dimensionless dielectric constant $\alpha _\epsilon = e^2/(4 \pi \epsilon _0 \epsilon _r) \, k_F^{(0)}/E^{(0)}\backsimeq 1.0$ was chosen in our computation, corresponding to $\epsilon _r = 2.4$ for $SiO_2$ substrate which is normally used for the plasmon calculation in graphene^19^. The role of background dielectric constant $\epsilon _r$ is certainly shaping out the plasmon branch which is very well seen in the long-wave limit, where $\Omega _p \backsim \sqrt{\alpha _\epsilon } \backsim 1/\sqrt{\epsilon _r}$, i.e., a larger $\epsilon _r$ leads to a smaller plasmon $\Omega _p$ for fixed wave number $q_y$.

For a metallic ribbon with no gap and for a reasonably small ${\mathbb {N}}_R < 100$, we found that the plasmon dispersions remain the same for graphene and all other types of $\alpha -{\mathcal {T}}_3$ materials including a dice lattice. The one-subband model for the polarizability showing no doping or $\alpha$ dependence seems to work well for all such ribbons (zero gap and a relatively small width). In a wide ribbon, however, many subbands need to be taken into account since a few of them will be populated under electron doping. We also need to keep in mind that one cannot apply the Dirac Hamiltonian approximation for bulk to a very narrow ribbon so that the previously addressed situation with low doping and large subband separation is transparent but very limited and cannot be employed for most realistic problems. The situation becomes drastically different if a gap is present. Both inter- and intra-band portions of particle-hole modes are split into two separate regions in the $\omega$-$q_y$ plane and a strong dependence on $\alpha$ is observed irrespective of the ribbon width.

**Figure 6:**
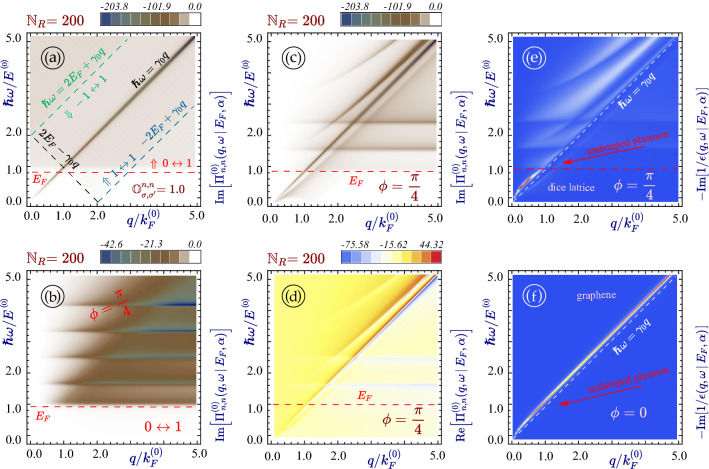
Intra-subband polarization function $\Pi ^{(0)}_{n,n}(q_y, \omega \, \vert \, E_F, \alpha )$, single-particle excitation spectrum and plasmon dispersion for a very wide metallic nanoribbon with ${\mathbb {N}}_R = 200$ and $N_0 = ({\mathbb {N}}_R + 1)/3 = 67$. Panel (**a**) presents a (unrealistic) case when all overlaps ${\mathbb {O}}^{\,n,n'}_{\sigma _1 \leftrightarrow \sigma _2} (k_y,q_y)$ ($\sigma _{1,2} = 0,\,\pm 1$) are set to unity with an equal contribution to $\Pi ^{(0)}_{n,n'}(q_y, \omega \, \vert \, E_F, \alpha )$. Panel (**b**) shows the contribution of transitions from and to the flat band *only*, i.e., setting ${\mathbb {O}}^{n,n}_{\,\pm 1 \leftrightarrow \pm 1} (k_y,q) = 0$, for a dice lattice with $\phi = \pi /4$. Panels (**c**) and (**d**) display the imaginary and real parts of $\Pi ^{(0)}_{n,n}(q_y, \omega \, \vert \, E_F, \alpha )$ for a dice lattice when all nine intra-band and inter-band transitions are included. Panels (**e**) and (**f**) exhibit the calculated plasmon dispersions by tracking peaks of a spectral-loss function ${\mathbb {S}}(q_y, \omega \, \vert \, E_F, \alpha )$ for a dice lattice and graphene with $\phi =\pi /4$ and $\phi =0$, separately. Other parameters are the same as those in Fig. 2.

Figure 6 demonstrates the effect due to relative hopping played by $\alpha$ in a very wide nanoribbon with ${\mathbb {N}}_R = 200$. The separation between two adjacent subbands decreases to $\backsim 0.7 E^{(0)}$ in order to occupy multiple subbands for chosen doping to approach bulk material. As a starting point, we first examine a (unrealistic) situation, as in Fig. 6a, by setting all overlaps to unity. In this case, we are able to see contributions from all transitions as well as a strong dissipation peak near the main diagonal $\hbar \omega = \gamma _0 q$.

When only the flat band $0\leftrightarrow 1$ transitions are considered, we expect Landau damping of plasmons due to the presence of particle-hole modes within the region $\hbar \omega \ge E_F$, as seen in Fig. 6b, which appears as a layer structured because of the nature of discrete subbands. Additionally, its contribution to ${\text {Im}}\left[ \Pi ^{(0)}_{n,n}(q_y, \omega ) \right]$ at $\hbar \omega =E_F$ is about half its maximum, and it increases with $\omega$ since more subband contributions are included. Meanwhile, the flat-band dissipation becomes stronger with increasing wave vector $q_y$ for chosen $\omega$. Such strong plasmon damping at relatively low $q_y$ is responsible for the significant change in plasmon dispersions from graphene to $\alpha -{\mathcal {T}}_3$ for both bulk and nanoribbon, as can be easily verified from Fig. 6e and f, where the plasmon branch also exhibits a unique shape for pinching when $\hbar \Omega _p(q_y)/E_F=1$ at the point where $q_y/k_F^{(0)}=1$. Specifically, for a dice lattice we display both its plasmon dispersion in Fig. 6d and dissipation in Fig. 6c, where all intra-subband and inter-subband transitions among nine subbands have been considered. The trace of Landau damping of plasmons from flat-band $0\leftrightarrow 1$ transitions is clearly visible in Fig. 6c, in addition to plasmon damping due to intra-subband and inter-subband transitions.

**Figure 7:**
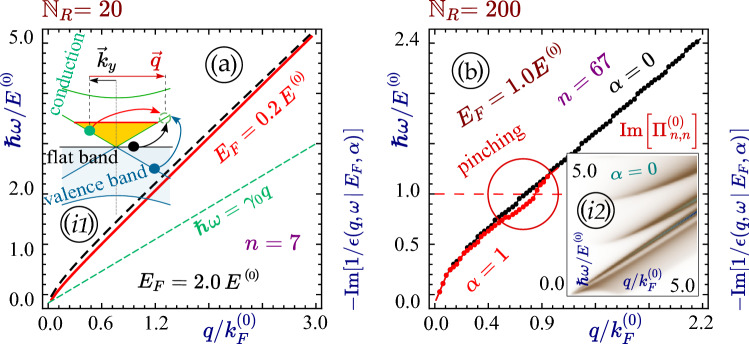
Panel (**a**) presents plasmons for different electron densities, correspond to $E_F/E^{(0)}=0.2$ (black) and 2.0 (red) in a narrow ribbon of ${\mathbb {N}}_R = 20$. Panel (**b**) displays plasmons for graphene (black) and a dice lattice (red) in a wide nanoribbon of ${\mathbb {N}}_R = 200$ for $E_F/E^{(0)}=1.0$. Inset (*i*1) in (**a**) illustrates all allowed transitions in a narrow metallic electron-doped $\alpha -{\mathcal {T}}_3$ ribbon, while inset (*i*2) in (**b**) exhibits single-particle excitation spectrum in a wide metallic graphene nanoribbon. Other parameters are the same as those in Fig. 2.

We find that the nanoribbon plasmon frequency is much less sensitive to the electron doping or Fermi energy $E_F$ than in the case of bulk graphene (or a dice lattice), as demonstrated by the red-solid and black-dashed curves in Fig. 7a. As we showed above, the polarization function is completely independent of $E_F$ in one-subband approximation which represents the dominant contribution for narrow ribbons with metallic dispersions. In the general case, the polarization function becomes independent of $E_F$ if $q > E_F/\gamma _0$, as explained by inset (*i*1) of Fig. 7a. At the same time, the strength of pinching in a wide nanoribbon can be enhanced by increasing $\alpha$ from zero to unity, as seen in Fig. 7b.

## Summary and remarks

In this paper, we have calculated the dynamical polarization function, dielectric tensor, the resulting plasmon dispersions and Landau damping for various types of $\alpha -{\mathcal {T}}_3$ nanoribbons with armchair edge termination. Armchair nanoribbons are distinguished because their longitudinal $k_y$ and transverse $\xi _n$ components of the electron momentum are not coupled to each other, the wave functions and their overlaps are similar to those for the bulk except for the quantized $\xi _n$ and the energy dispersions, as well as the valley mixing.

The plasmon excitations in a nanoribbon and their Landau damping (single-particle excitation spectrum) are mainly determined by the energy bandgap in the electron dispersions which directly depend on the width of the ribbon or, specifically, on the number ${\mathbb {N}}_R$ of atomic rows across the ribbon. The ribbon is metallic (zero bandgap between the valence, flat and conduction bands) if ${\mathbb {N}}_R + 1$ is an integer multiple of 3. For all other numbers ${\mathbb {N}}_R$, the ribbon has a $\backsim 1/{\mathbb {N}}_R$ gap even though the bulk bandstructure is metallic which holds true for the ribbons made of all $\alpha -{\mathcal {T}}_3$ materials including graphene and dice.

The main focus of our investigation has been to identify the role of the relative hopping parameter $\alpha$ or the distinctions between the plasmon modes in nanoribbons based on various $\alpha -{\mathcal {T}}_3$ materials. These materials, specifically a dice lattice, stand out due to the presence of an additional dispersionless zero-energy band in the electron dispersions, and the electron transitions to and from this flat band. The overlaps of the transitions between the valence and partially occupied conduction band are also affected by $\alpha$. An additional particle hole mode above the Fermi level is also observed for all nanoribbons with a finite gap, however, its lower boundary is shifted up to $E_F + \Delta _0$ since the energy separation between the zero-energy flat band and the Fermi energy should also include the gap.

The most substantial difference between bulk $\alpha -{\mathcal {T}}_3$ and nanoribbons stems from the quantization of the energy bandstructure. The discernible contribution from various energy subbands is clearly seen in separate regions of the single-particle excitation spectrum. Another feature of a nanoribbon is that the overlaps only depend on a single-subband index *n*, in a narrow ribbon, they become either equal or close to zero for some transitions which otherwise would be dominant in a bulk material so that in many cases the terms contributing to the polarization disappear. Finally, the dielectric tensor of a nanoribbon is such that only intra-subband ($n \leftrightarrow n$) transitions need to be included in the polarizability.

In a nutshell, we have performed a comprehensive study on the plasmon excitations in an $\alpha -{\mathcal {T}}_3$ nanoribbon. We discovered some very distinctive features of plasmon dispersion and the distribution of its finite Landau damping with respect to the width of a ribbon, absence/presence of a bandgap, electron doping density and a phase of the $\alpha -{\mathcal {T}}_3$ lattice. These previously unreported plasmonic properties have the potential of being widely employed in next-generation nanoribbon-based electronic, optical and plasmonic quantum devices.

## Supplementary Information

Supplementary Information.
