# Supplementary material for: Tailoring plasmon excitations in α - 𝒯3 armchair nanoribbons
Source: Sci Rep. 2021 Oct 18;11:20577. doi: 10.1038/s41598-021-99596-z (PMC8523740; doi:10.1038/s41598-021-99596-z)
Supplement: Supplementary file 1 — Supplementary Information. [file 41598_2021_99596_MOESM1_ESM.pdf]

SUPPLEMENTARY INFORMATION to
Tailoring plasmon excitations in $\alpha - \mathcal{T}_3$ armchair nanoribbons

(Dated: September 22, 2021)

I. PHASE-DEPENDENT ELECTRONIC STATES IN NANORIBBONS: DERIVATION OF EQS. (2), (7) AND (9).

The case for a finite-width nanoribbon is distinguished from a bulk $\alpha - \mathcal{T}_3$ model since the $\mathbf{K}$ and $\mathbf{K}'$ valleys are mixed and the total Hamiltonian should include both of them

$$\mathbb{H}_{all}^{\phi}(\boldsymbol{k}) = \left\{ \begin{array}{c|c} \mathbb{H}_r^{\tau=1,\,\phi}(\boldsymbol{k}) & 0 \\ \hline 0 & \mathbb{H}_r^{\tau=-1,\,\phi}(\boldsymbol{k}) \end{array} \right\} \, , \tag{1}$$

as well as due to absence of translational symmetry in the transverse (x) direction so that the replacement of $k_x \longrightarrow -i\partial/\partial x$, and therefore the Hamiltonian in Eq. [1] is modified into

$$\mathbb{H}_r^{\tau,\,\phi}(\boldsymbol{k}) = \gamma_0 \left\{ \begin{array}{ccc} 0 & (-i\tau\partial/\partial x - ik_y)\,\cos\phi & 0 \\ (-i\tau\partial/\partial x + ik_y)\,\cos\phi & 0 & (-i\tau\partial/\partial x - ik_y)\,\sin\phi \\ 0 & (-i\tau\partial/\partial x + ik_y)\,\sin\phi & 0 \end{array} \right\} \, , \tag{2}$$

which implies that the transverse momentum k_x in the system is no longer a good quantum number anymore.

The boundary conditions for wave function are the same as those of a dice lattice by requiring that all three sublattice probability currents, including one for H atom, disappear at each boundary of a nanoribbon. This gives rise to

$$\begin{aligned} &\varphi_\nu(x)\big|_{x=0} = \varphi'_\nu(x)\big|_{x=0} \, , \\ &\varphi_\nu(x)\big|_{x=W_R} = \mathsf{exp}(i\delta K_x W_R)\, \varphi'_\nu(x)\big|_{x=W_R} \, , \end{aligned} \tag{3}$$

where $\nu = A$, B and H, $\varphi_\nu(x)$ and $\varphi'_\nu(x)$ correspond to wave-function components belonging to $\boldsymbol{K}$ and $\boldsymbol{K}'$ valleys, respectively. From Eq. (3) it is clear that the boundary conditions have mixed the electronic states from both $\boldsymbol{K}$ and $\boldsymbol{K}'$ valleys, similar to graphene and therefore both valleys need to be considered. In addition, the wave functions associated with the Hamiltonian in Eq. (2) take the form

$$\Phi_\sigma^\phi(n\,|\,x,k_y) = \left[\begin{array}{c} \Psi_\sigma^{\mathbf{K},\phi}(n\,|\,x,k_y) \\ \Psi_\sigma^{\mathbf{K}',\phi}(n\,|\,x,k_y) \end{array} \right] \, , \tag{4}$$

where each one of two components in Eq. (4) is assigned to a specific valley ($\tau = \pm 1$) and formally written as

$$\Psi_\sigma^{\mathbf{K},\phi}(n\,|\,x,k_y) = \left[\begin{array}{c} \varphi_A(x\,|\,\phi,n) \\ \varphi_H(x\,|\,\phi,n) \\ \varphi_B(x\,|\,\phi,n) \end{array} \right] \mathsf{e}^{ik_y y} \, . \tag{5}$$

The remaining components of eigenstates in Eqs. (5) could be determined by Hamiltonian in Eq. (2). Specifically, for the hub state $\varphi_H(x)$, we have

$$\frac{1}{2}\, k_\varepsilon^2\, \varphi_H(x) = - \left[\frac{\partial^2}{\partial x^2} - k_y^2 \right] \varphi_H(x) \, , \tag{6}$$

leading to the solution

$$\varphi_H(x) = A\,\mathrm{e}^{i\xi x} + B\,\mathrm{e}^{-i\xi x}\ , \tag{7}$$

where $\xi^2 = k_\varepsilon^2 - k_y^2$, $k_\varepsilon = \varepsilon/\gamma_0$ and ε is the energy of electrons. Considering the fact that the wave function corresponding to each valley has only one direction for the transverse wave number ξ , i.e. $\mathrm{e}^{+i\xi x}$ for $\boldsymbol{K}$ while $\mathrm{e}^{-i\xi x}$ for $\boldsymbol{K}'$, we can set $\varphi_H(x) = A\,\mathrm{e}^{i\xi x}$ and $\varphi'_H(x) = A'\,\mathrm{e}^{-i\xi x}$. As a result, the boundary conditions in Eq. (3) yield

$$\begin{aligned} & A - B' = 0\ , \\ & A\,\mathrm{e}^{i\xi W_R} - \mathrm{e}^{i\delta K_x W_R}\,B'\,\mathrm{e}^{-i\xi W_R} = 0\ , \end{aligned} \tag{8}$$

which acquires a non-trivial solutions only if

$$\mathrm{e}^{i\xi W_R} - \mathtt{exp}(i\delta K_x W_R)\,\mathrm{e}^{-i\xi W_R} = 0\ . \tag{9}$$

Since W_R is arbitrarily, Eq. (9) leads to

$$2\xi W_R = 2\pi n + \delta K_x W_R\ , \qquad\qquad n = 0,\ \pm1,\ \pm2,\ \pm3,\ \cdots\ . \tag{10}$$

It should be mentioned that the quantization condition (10) for transverse momentum ξ_n could be immediately obtained from the second boundary condition (3) (for $x = W_R$) for the $H-$components of the wave function in two different valleys: $\varphi_H(x) \backsim \mathrm{e}^{i\xi x}$ and $\varphi'_H(x) \backsim \mathrm{e}^{-i\xi x}$ (the constants will be later found from the normalization) as

$$\mathrm{e}^{i\xi W_R} = \mathtt{exp}(i\delta K_x W_R)\,\mathrm{e}^{-i\xi W_R}\ , \tag{11}$$

which is equivalent to Eq. (24) and is the same for graphene, a dice lattice and, as an interpolation, for all $\alpha - \mathcal{T}_3$ materials. This is the most crucial part of determining the nanoribbon wave function since the relation between the components of (4) are the same as in the bulk.

Once $\varphi_H(x)$ is obtained from Eq. (6), we can easily find the remaining two components of the transverse wave functions introduced in Eq. (5), yielding

$$\begin{aligned} \varphi_A(x) &= \frac{1}{k_\varepsilon}\left(-i\tau\frac{\partial}{\partial x} - ik_y\right)\cos\phi\,\varphi_H(x)\ , \\ \varphi_B(x) &= \frac{1}{k_\varepsilon}\left(-i\tau\frac{\partial}{\partial x} + ik_y\right)\sin\phi\,\varphi_H(x)\ , \end{aligned} \tag{12}$$

where $\tau = \pm1$ correspond to two different valleys. In general, we can write down the full expression of wave function by

$$\Psi^{\tau,\phi}_{\sigma=\pm1}(n\,|\,x, k_y) = \frac{1}{\sqrt{\mathcal{L}_0 W_R}}\,\varphi^{\tau,\phi}_{\sigma=\pm1}(n\,|\,x)\,\mathrm{e}^{ik_y y}\ , \tag{13}$$

where $\mathcal{L}_0$ is the length of a ribbon,

$$\varphi^{\tau,\phi}_{\sigma=\pm1}(n\,|\,x) = \frac{1}{\sqrt{2}}\begin{bmatrix} \tau\cos\phi\,\mathrm{e}^{-i\tau\Theta_n(k_y)} \\ \sigma \\ \tau\sin\phi\,\mathrm{e}^{i\tau\Theta_n(k_y)} \end{bmatrix}\mathtt{exp}(i\tau\xi_n x)\ . \tag{14}$$

and $\Theta_n(k_y) = \tan^{-1}(k_y/\xi_n)$. It is crucial to notice that only the electron/hole index $\sigma = \pm1$, not the subband index n, determines the $\pm$ sign of energy dispersions $\varepsilon^\sigma_n(k_y)$. Since Eq. (6) allows for an additional solutions with $k_\varepsilon = 0$, for a zero-energy flat band, on the other hand, we have

$$\left(\frac{\partial}{\partial x} - k_y\right)\,\cos\phi\,\varphi_A(x) + \left(\frac{\partial}{\partial x} + k_y\right)\,\sin\phi\,\varphi_B(x) = 0\ , \tag{15}$$

$$\left(-\frac{\partial}{\partial x} - k_y\right)\,\cos\phi\,\varphi'_A(x) + \left(-\frac{\partial}{\partial x} + k_y\right)\,\sin\phi\,\varphi'_B(x) = 0\ , \tag{16}$$

from which we see that these two equations valley differ only by the sign of x-derivative $\partial/\partial x$ for $\boldsymbol{K}$ and $\boldsymbol{K}'$ valleys.

Here, two equations in Eq. (15) have simple solutions in the form of $\varphi_\nu(x) \backsim \mathsf{e}^{\pm i\zeta x}$. However, each valley only support one direction for the transverse wave number ξ, i.e., $\backsim \mathsf{e}^{+i\zeta x}$ for $\boldsymbol{K}$ valley whereas $\backsim \mathsf{e}^{-i\zeta x}$ for $\boldsymbol{K}'$ valley. Correspondingly, we set $\varphi_A(x) = A\mathsf{e}^{i\zeta x}$, $\varphi_B(x) = B\mathsf{e}^{i\zeta x}$, and $\varphi'_A(x) = A'\mathsf{e}^{i\zeta x}$, $\varphi'_B(x) = B'\mathsf{e}^{i\zeta x}$. As a result, for $0 < x < W_R$ Eq. (15) could be simply rewritten as

$$\begin{aligned} &\cos\phi\,\left(-i\zeta + k_y\right)\,A + \sin\phi\,\left(-i\zeta + k_y\right)\,B = 0\ , \\ &\cos\phi\,\left(+i\zeta - k_y\right)\,A' + \sin\phi\,\left(+i\zeta + k_y\right)\,B' = 0\ . \end{aligned} \tag{17}$$

For two boundaries at $x = 0$ and $x = W_R$, we get from the boundary conditions in Eq. (3) that

$$A - A' = 0\ , \tag{18}$$

$$B - B' = 0\ , \tag{19}$$

$$A\,\mathsf{e}^{+i\xi W_R} - \mathsf{e}^{i\delta K_x\,W_R}\,A'\,\mathsf{e}^{-i\xi W_R} = 0\ , \tag{20}$$

$$B\,\mathsf{e}^{+i\xi W_R} - \mathsf{e}^{i\delta K_x\,W_R}\,B'\,\mathsf{e}^{-i\xi W_R} = 0\ . \tag{21}$$

which makes our system over determined. However, Eqs. (17), (18) and (20) are compatible, leading to a non-zero solution only if

$$-\sin^2\phi\,\left[\mathsf{e}^{+i\xi W_R} - \mathsf{exp}(i\delta K_x W_R)\,\mathsf{e}^{-i\xi W_R}\right]\,\left(k_y^2 + \xi^2\right) = 0 \tag{22}$$

is satisfied. Since k_y could be arbitrary, this gives rise to the following condition

$$\xi W_R = 2\pi n + \delta K_x W_R - \xi W_R\ , \qquad\qquad n = 0,\ \pm1,\ \pm2,\ \pm3,\ \cdots\ , \tag{23}$$

or equivalently,

$$\xi_n = \frac{\pi n}{W_R} + \frac{1}{2}\,\delta K_x\ , \tag{24}$$

which turns out the same as that in Eq. (10). Eventually, the wave function for the flat band with $\sigma = 0$ takes the form

$$\Psi^{\tau,\phi}_{\sigma=0}(n\,|\,x, k_y) = \frac{1}{\sqrt{\mathcal{L}_0 W_R}}\ \varphi^{\tau,\phi}_{\sigma=0}(n\,|\,x)\,\mathsf{e}^{ik_y y}\ , \tag{25}$$

where

$$\varphi^{\tau,\,\phi}_{\sigma=0}(n\,|\,x) = \begin{bmatrix} \left(\tau\xi_n - ik_y\right)\,\sin\phi \\ 0 \\ -\left(\tau\xi_n + ik_y\right)\,\cos\phi \end{bmatrix} \frac{\mathsf{e}^{i\tau\xi_n x}}{k_\varepsilon} = \begin{bmatrix} \tau\sin\phi\,\mathsf{e}^{-i\tau\Theta_n(k_y)} \\ 0 \\ -\tau\cos\phi\,\mathsf{e}^{i\tau\Theta_n(k_y)} \end{bmatrix} \mathsf{exp}(i\tau\xi_n x)\ , \tag{26}$$

in which $\boldsymbol{K}$ and $\boldsymbol{K}'$ valleys correspond to $\tau = \pm1$ and $\Theta_n(k_y) = \tan^{-1}(k_y/\xi_n)$.

II. POLARIZATION FUNCTION, PLASMON DISPERSIONS AND DAMPING DERIVATION OF EQ. (16).

A close look at the dielectric-function matrix in Eq. [15] reveals that its matrix elements depend only on the row index ν, implying all the off-diagonal elements in the same row are identical. Therefore, the determinant of matrix takes the form

$$\mathcal{D}et\,\left[\delta_{\mu,\nu}-V_0^0(q_y)\,\Pi^{(0)}_{\nu,\nu}(q_y,\omega\,|\,E_F,\alpha)\right] \\ =\sum_{\mu_1,\mu_2,\dots,\,\mu_{3N}=1}^{3N}\mathcal{L}_\varepsilon(\mu_1,\mu_2,\dots,\,\mu_{3N})\,\epsilon_{\mu\nu}(q_y,\omega\,|\,E_F,\alpha)_{1,\mu_1}\cdots\epsilon_{\mu\nu}(q_y,\omega\,|\,E_F,\alpha)_{3N,\,\mu_{3N}}\ , \tag{27}$$

where $\mathcal{L}_\varepsilon(\mu_1,\mu_2,\dots,\,\mu_{3N})$ is the Levi-Civita tensor, and this result can be further simplified to a single summation over the remaining composite index $\nu=\{j,\sigma_\nu\}$. This gives rise to

$$\mathcal{D}et\,\left[\epsilon_{\mu,\nu}(q_y,\omega\,|\,E_F,\alpha)\right]=1-V_0^0(q_y)\sum_{j=1}^{N}\Pi^{(0)}_{j,j}(q_y,\omega\,|\,E_F,\alpha)\,, \tag{28}$$

where $\Pi^{(0)}_{j,j}(q_y,\omega\,|\,E_F,\alpha)$ represents the jth subband polarizability which has already been summed over the valence, conductance and flat bands, i.e.

$$\Pi^{(0)}_{j,j}(q_y,\omega\,|\,E_F,\alpha)=\sum_{\sigma_\nu=0,\pm1}\Pi^{(0)}_{\nu,\nu}(q_y,\omega\,|\,E_F,\alpha)\ . \tag{29}$$

As an example, for $j=1,2$ we find

$$\mathcal{D}et\left[\begin{array}{cc}1-V_0^0(q_y)\,\Pi^{(0)}_{1,1}(q_y,\omega) & -V_0^0(q_y)\,\Pi^{(0)}_{2,2}(q_y,\omega)\\ -V_0^0(q_y)\,\Pi^{(0)}_{1,1}(q_y,\omega) & 1-V_0^0(q_y)\,\Pi^{(0)}_{2,2}(q_y,\omega)\end{array}\right] \\ =\left[1-V_0^0(q_y)\Pi^{(0)}_{1,1}\right]\left[1-V_0^0(q_y)\Pi^{(0)}_{2,2}\right]-\left[V_0^0(q_y)\right]^2\Pi^{(0)}_{1,1}\Pi^{(0)}_{2,2}=1-V_0^0(q_y)\left[\Pi^{(0)}_{1,1}+\Pi^{(0)}_{2,2}\right]\ , \tag{30}$$

which agrees with Eqs. (28) and (29).

Mathematically, the relation presented in Eq. (28) for a general $N\times N$ matrix could be proven by using the method of induction. We start with the Laplace expansion of the matrix over its last row

$$\left\{-V_0^0(q)\,\Pi^{(0)}_{3N,3N},\,-V_0^0(q)\,\Pi^{(0)}_{3N,3N}\,\cdots\,1-V_0^0(q)\,\Pi^{(0)}_{3N,3N}\right\}\,, \tag{31}$$

yielding

$$\mathcal{D}et\,\left[\epsilon^\mu_\nu(q_y,\omega)\right]=\sum_{\mu=1}^{3N}(-1)^{\mu+3N}\epsilon^\mu_{3N}\,\mathcal{M}_{\mu,3N} \\ =V_0^0(q_y)\,\sum_{\mu=1}^{3N-1}(-1)^{\mu+3N}V_0^0(q_y)\,\Pi^{(0)}_{\mu,\mu}\,\mathcal{M}_{\mu,3N}+\left[1-V_0^0(q_y)\,\Pi^{(0)}_{3N,3N}\right]\mathcal{M}_{3N,3N}\ , \tag{32}$$

where the corresponding minor matrices are given by

$$\mathcal{M}_{\mu,3N}=-V_0^0(q_y)\,\Pi^{(0)}_{\mu,3N}(q_y,\omega)\qquad\text{for}\qquad \mu\neq 3N \tag{33}$$

$$\mathcal{M}_{3N,3N}=1-V_0^0(q_y)\,\sum_{\mu=1}^{3N-1}\Pi^{(0)}_{\mu,3N}(q_y,\omega)\qquad\text{for}\qquad \mu=3N\ . \tag{34}$$

The validity of Eq. (34) is taken as the base for induction. The full summation in Eq. (32) amounts to Eq. (28). However, we must say that a complete proof of Eq. (28) falls off the scope of current paper.

III. ANALYTICAL EXPRESSION FOR THE POLARIZATION FUNCTION OBTAINED USING SINGLE SUBBAND APPROXIMATION FOR A LOW-DOPED METALLIC ANR DERIVATION OF EQS. (23), (25) AND (26).

First, we should mention that in calculating the polarization function we only consider the electron transitions between the valence and conduction bands similarly to graphene. All the additional terms associated with the flat band $0 \leftrightarrow 1$ and $1 \leftrightarrow 0 \backsim \sin[\Theta_{n,n'}(k_y, q_y)]$ are equal to zero if $\xi_n = 0$ and need to be excluded from our consideration.

At zero temperature, the Fermi distribution functions are simplified to Heaviside step functions $\Theta(x)$ as

$$f_0[\varepsilon_\mu(k_y)\,|\,u_0(T), T \to 0] = \left\{ 1 + \texttt{exp}\left[\frac{\varepsilon_\mu(k_y) - E_F}{k_B T} \right] \right\}^{-1} \Bigg|_{T\to 0} \to \delta_{\sigma,-1} + \delta_{\sigma,1}\,\Theta[E_F - \varepsilon_\mu(k_y)]\;, \tag{35}$$

and the polarizability could be written as a sum of three separate terms related to the inter- and intra-band electron transitions

$$\Pi^{(0)}(q,\omega) = -\chi_\infty^{(-)}(q,\omega) + \sum_{\beta=\pm 1} \chi_{E_F}^{(\beta)}(q,\omega)\,. \tag{36}$$

Here, the first term $\chi_\infty^{(-)}(q,\omega)$ corresponds to the zero doping and is equal to

$$\chi_\infty^{(-)}(q,\omega) = g_s \sum_{\beta=\pm 1} \int \frac{dk_y}{2\pi} \frac{\mathbb{O}^{N_0,N_0}_{-1\leftrightarrow +1}(k_y, k_y+q)}{\varepsilon_{N_0}(k_y) + \varepsilon_{N_0}(k_y+q) + \beta\,\omega}\,. \tag{37}$$

The two remaining terms of Eq. 36 which appear only for a finite doping are

$$\chi_{E_F}^{(\rho=\pm)}(q,\omega) = g_s \sum_{\beta=\pm} \int \frac{dk_y}{2\pi}\, \mathbb{O}^{N_0,N_0}_{\rho\leftrightarrow +1}(k_y, k_y+\beta q)\, \frac{\Theta\left[E_F - \varepsilon_{N_0}(k_y)\right]}{\varepsilon_{N_0}(k_y) - \rho\,\varepsilon_{N_0}(k_y+\beta q) + \beta\,\omega} \tag{38}$$

or specifically,

$$\chi_{E_F}^{(-)}(q,\omega) = g_s \sum_{\beta=\pm 1} \int \frac{dk_y}{2\pi}\, \mathbb{O}^{N_0,N_0}_{-1\leftrightarrow +1}(k_y, k_y+\beta q)\, \frac{\Theta\left[E_F - \varepsilon_{N_0}(k_y)\right]}{\varepsilon_{N_0}(k_y) + \varepsilon_{N_0}(k_y+\beta q) + \beta\,\omega} \tag{39}$$

and

$$\chi_{E_F}^{(+)}(q,\omega) = g_s \sum_{\beta=\pm 1} \int \frac{dk_y}{2\pi}\, \mathbb{O}^{N_0,N_0}_{+1\leftrightarrow +1}(k_y, k_y+\beta q) \frac{\Theta\left[E_F - \varepsilon_{N_0}(k_y)\right]}{\varepsilon_{N_0}(k_y) - \varepsilon_{N_0}(k_y+\beta q) + \beta\,\omega}\,. \tag{40}$$

Each of the integrals in Eqs. (37) and (38) is easily evaluated because each of the overlaps $\mathbb{O}^{N_0,N_0}_{\sigma\leftrightarrow\sigma'}(k_y, k_y+\beta q)$ is equal either 0 or 1 as

$$\mathbb{O}^{N_0,N_0}_{\sigma\leftrightarrow\sigma'}(k_y, k_y+\beta q) = \delta_{\sigma\sigma',\,\text{sign}[k_y\cdot(k_y+\beta q)]}\,. \tag{41}$$

The only lengthy and tedious part of this derivation is the careful identification of the ranges of k_y in which the integrand is not zero. The complete schematics of these regions is presented n Fig. 1. One has to keep in mind multiple integration cut offs due to both a specific form of the overlaps (41) and due to the finite doping value E_F. The difference of energies in the denominator $\varepsilon_{N_0}(k_y) + \beta\varepsilon_{N_0}(k_y+\beta q) = \pm\gamma_0 q$ should be evaluated separately for each term of summation (36) but never depends on k_y.

Finally, zero-doping term (37) is evaluated as

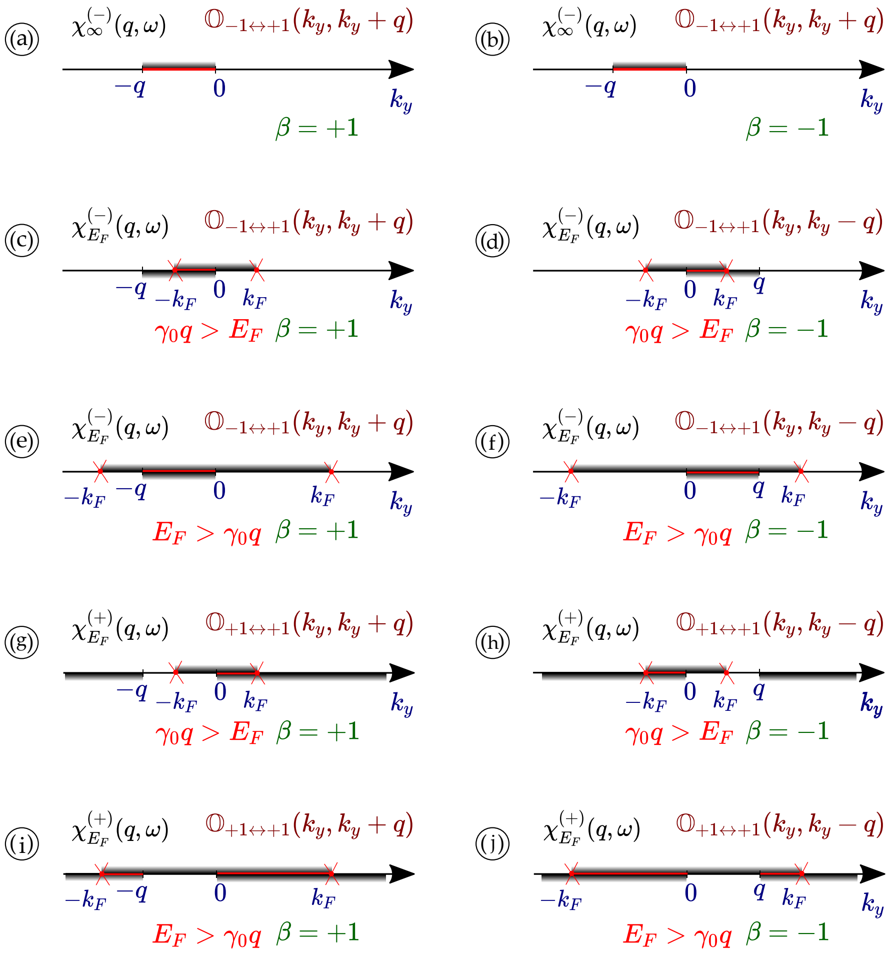

FIG. 1: (Color online) Schematics for all the regions of $\int dk_y$ with non-zero integrand functions for evaluating each term in Eqs. (37) and (38). Each left panel corresponds to the first term $\beta=1$ of the summation in each of these equations, the right ones - to $\beta=-1$. Expression (37) which is shown in plots (a) and (b) does not depend on the doping E_F and, therefore, there is no reason to consider the two separate cases of $E_F>\gamma_0 q$ and $E_F<\gamma_0 q$, as we did for all the remaining terms.

$$\chi_\infty^{(-)}(q,\omega) = -\frac{2\gamma_0}{\pi}\frac{q^2}{\omega^2-(\gamma_0 q)^2}\,, \tag{42}$$

and the other two terms of (36) are

$$\chi_{E_F}^{(-)}(q,\omega) = g_s\sum_{\beta=\pm}\int\frac{dk_y}{2\pi}\,\Theta\left[\frac{E_F}{\gamma_0}-|k_y|\right]\,\Theta[-\beta k_y(k_y+\beta q)]\,(\gamma_0 q+\beta\omega)^{-1} = -\frac{2\gamma_0 q}{\pi}\frac{\mathrm{Min}(q,k_F)}{\omega^2-(\gamma_0 q)^2}\,. \tag{43}$$

and

$$\chi_{E_F}^{(+)}(q,\omega) = \frac{2\gamma_0}{\pi}\frac{k_F\,q}{\omega^2-q^2}\left\{1-\left(1-\frac{q}{k_F}\right)\Theta[k_F-q]\right\}\,, \tag{44}$$

which is equivalent to

$$\chi_{E_F}^{(+)}(q,\omega) = \frac{2\gamma_0 q}{\pi}\frac{\mathrm{Min}(q,k_F)}{\omega^2-(\gamma_0 q)^2} = -\chi_{E_F}^{(-)}(q,\omega)\,, \tag{45}$$

which means that $\chi_{E_F}^{(+)}(q,\omega)+\chi_{E_F}^{(-)}(q,\omega)=0$ and the two finite-doping terms cancel each other.
